# Three E2F target-related genes signature for predicting prognosis, immune features, and drug sensitivity in hepatocellular carcinoma

**DOI:** 10.3389/fmolb.2023.1266515

**Published:** 2023-10-03

**Authors:** Baozhu Zhang, Boyang Chang, Lu Wang, Yuzhong Xu

**Affiliations:** ^1^ Department of Radiation Oncology, The People’s Hospital of Baoan Shenzhen, The Second Affiliated Hospital of Shenzhen University, Shenzhen, China; ^2^ Department of Interventional Radiology, The Third Affiliated Hospital of Sun Yat-Sen University, Guangzhou, China; ^3^ Department of Clinical Laboratory, The People’s Hospital of Baoan Shenzhen, The Second Affiliated Hospital of Shenzhen University, Shenzhen, China

**Keywords:** E2F target, hepatocellular carcinoma, prognosis, immune landscape, drug sensitivity

## Abstract

**Background:** Hepatocellular carcinoma (HCC) is extremely malignant and difficult to treat. The adenoviral early region 2 binding factors (E2Fs) target pathway is thought to have a major role in tumor growth. This study aimed to identify a predictive E2F target signature and facilitate individualized treatment for HCC patients.

**Methods:** We constructed an E2F target-related gene profile using univariate COX and LASSO regression models and proved its predictive efficacy in external cohorts. Furthermore, we characterized the role of the E2F target pathway in pathway enrichment, immune cell infiltration, and drug sensitivity of HCC.

**Results:** Lasso Cox regression created an E2F target-related gene signature of GHR, TRIP13, and CDCA8. HCC patients with high risk were correlated with shorter survival time, immune evasion, tumor stem cell characteristics and high sensitivity to Tipifarnib and Camptothecin drugs.

**Conclusion:** Hepatocellular carcinoma prognosis was predicted by an E2F target signature. This finding establishes the theoretical usefulness of the E2F target route in customized identification and treatment for future research.

## Introduction

Liver cancer is the third leading cause of cancer-related death worldwide, with an expected 830,000 fatalities in 2020 (Sung et al., 2021). Hepatocellular carcinoma (HCC) accounts for over three-quarters of liver cancer cases. Two-thirds of patients are diagnosed when they cannot undergo curative surgery. Therefore, early diagnosis is necessary to improve the therapeutic effects and prognosis of HCC. Exploring novel genes and pathways is urgent to promote early diagnosis and individualized treatment.

The cyclin-dependent kinase (CDK), retinoblastoma transcriptional corepressor 1 (RB1), and the adenoviral early region 2 binding factors (E2Fs) form a sophisticated mechanism to control cell cycle progression (Kent and Leone, 2019). E2Fs detach from the E2F-RB1 complex and promote cell cycle-dependent gene transcription when RB1 is altered or phosphorylated (van den Heuvel and Dyson, 2008; Zheng et al., 2023). Upregulation of E2F target genes is correlated with poor outcomes of neuroblastoma, breast cancer, colorectal cancer, ovarian cancer, and prostate cancer (Molenaar et al., 2012; Dahl et al., 2019; Oshi et al., 2021; Xia et al., 2022; Xu et al., 2022). Moreover, the E2F score serves as a predictive biomarker of response to neoadjuvant chemotherapy in estrogen receptor (ER)-positive/human epidermal growth factor receptor 2 (HER2)-negative breast cancer patients (Oshi et al., 2020).

A gene signature of the E2F target pathway was created to predict HCC patients’ prognoses. First, using single-sample gene set enrichment analysis (ssGSEA), we identified that the E2F target pathway affects HCC prognosis. Weighted gene co-expression network analysis (WGCNA) and differential expression gene (DEG) analysis were used to uncover the E2F target-related gene set related to HCC prognosis. Using The Cancer Genome Atlas (TCGA) data as a training group, we found an E2F target-related gene signature using univariate analysis and least absolute shrinkage and selection operator (LASSO) Cox regression analyses. Each patient’s E2F target pathway risk score was calculated based on the established gene signature.

Additionally, two external cohorts from the International Cancer Genome Consortium (ICGC) and Gene Expression Omnibus (GEO) were used to validate the predictive power of the E2F target gene signature. We also compared clinical characteristics, route enrichment, immune cell infiltration, and medication sensitivity across risk groups. The novel E2F target gene signature could contribute to the judgment of patients’ prognosis and guidelines of clinical therapy for HCC patients.

## Materials and methods

### Data acquisition and processing

Six hundred forty-three HCC patients from TCGA, GEO, and ICGC were enrolled. A total of 370 HCC patients with comprehensive transcriptional and clinical data from the TCGA database (https://portal.gdc.cancer.gov/) were used as a training cohort. In this study, two external cohorts of HCC were used as validation groups. In validation group 1, 243 HCC patients were selected from the ICGC database (https://dcc.icgc.org/projects/). The validation group 2 consists of 30 HCC patients from the GSE107943 database (https://www.ncbi.nlm.nih.gov/geo/query/acc.cgi?acc= GSE107943).

### Construction of E2F target signature

Reference genes were chosen from the Molecular Signatures Database (MSigDB) (http://www.gsea-msigdb.org/gsea/msigdb/search.jsp) (Table 1).

**TABLE 1:** HALLMARK_E2F_TARGETS (Number:200).

Gene name
AK2 ANP32E ASF1A ASF1B ATAD2 AURKA AURKB BARD1 BIRC5 BRCA1 BRCA2 BRMS1L BUB1B CBX5 CCNB2 CCNE1 CCP110 CDC20 CDC25A CDC25B CDCA3 CDCA8 CDK1 CDK4 CDKN1A CDKN1B CDKN2A CDKN2C CDKN3 CENPE CENPM CHEK1 CHEK2 CIT CKS1B CKS2 CNOT9 CSE1L CTCF CTPS1 DCK DCLRE1B DCTPP1 DDX39A DEK DEPDC1 DIAPH3 DLGAP5 DNMT1 DONSON DSCC1 DUT E2F8 EED EIF2S1 ESPL1 EXOSC8 EZH2 GINS1 GINS3 GINS4 GSPT1 H2AX H2AZ1 HALLMARK_E2F_TARGETS HELLS HMGA1 HMGB2 HMGB3 HMMR HNRNPD HUS1 ILF3 ING3 IPO7 JPT1 KIF18B KIF22 KIF2C KIF4A KPNA2 LBR LIG1 LMNB1 LUC7L3 LYAR MAD2L1 MCM2 MCM3 MCM4 MCM5 MCM6 MCM7 MELK MKI67 MLH1 MMS22L MRE11 MSH2 MTHFD2 MXD3 MYBL2 MYC NAA38 NAP1L1 NASP NBN NCAPD2 NME1 NOLC1 NOP56 NUDT21 NUP107 NUP153 NUP205 ORC2 ORC6 PA2G4 PAICS PAN2 PCNA PDS5B PHF5A PLK1 PLK4 PMS2 PNN POLA2 POLD1 POLD2 POLD3 POLE POLE4 POP7 PPM1D PPP1R8 PRDX4 PRIM2 PRKDC PRPS1 PSIP1 PSMC3IP PTTG1 RACGAP1 RAD1 RAD21 RAD50 RAD51AP1 RAD51C RAN RANBP1 RBBP7 RFC1 RFC2 RFC3 RNASEH2A RPA1 RPA2 RPA3 RRM2 SHMT1 SLBP SMC1A SMC3 SMC4 SMC6 SNRPB SPAG5 SPC24 SPC25 SRSF1 SRSF2 SSRP1 STAG1 STMN1 SUV39H1 SYNCRIP TACC3 TBRG4 TCF19 TFRC TIMELESS TIPIN TK1 TMPO TOP2A TP53 TRA2B TRIP13 TUBB TUBG1 UBE2S UBE2T UBR7 UNG USP1 WDR90 WEE1 XPO1 XRCC6 ZW10

We calculated GSEA in the training group using the R package “GSVA” based on the above-mentioned gene set (Lee et al., 2008). R package for WGCNA was used to evaluate TCGA database mRNA matrix data (Langfelder and Horvath, 2008). We generated an adjacency matrix and then converted it into a topological overlap matrix (TOM) to assess the correlation strength between the nodes. By performing hierarchical clustering, we ensured that each module contained at least 50 genes. Finally, we integrated similar modules to find module genes having a good correlation with ssGSEA E2F target scores. By combining these genes with the E2F target gene set in HALLMARKS, we got a new gene set named gene set A.

Univariate Cox regression was used to identify gene set B’s prognosis-related genes from gene set A. We used R package “limma” to determine DEGs between TCGA-LIHC tumors and healthy controls with a false discovery rate [FDR] < 0.05 and a log|fold change [FC]|> 1) (Ritchie et al., 2015). E2F-related DEGs and gene set B were intersected to obtain candidate genes to construct the gene signature. To avoid overfitting, LASSO Cox regression (R package “glmnet”) was conducted to exclude collinear genes (T and ibshirani, 1997). Finally, the three best model genes were selected, and their coefficients were recorded to create an E2F target gene signature.

### Subtypes based on etiology of the TCGA HCC cohorts

According to the contents of the “hist_hepato_carc_fact” record in the clinical information provided in the UCSC database, liver cancer samples were divided into HBV, HCV, ALD, and NASH. Finally, 104 samples with Hepatitis B, 56 samples with Hepatitis C, and 20 samples with Non-Alcoholic Fatty Liver Disease were retrieved. Alcoholic liver disease (ALD) related content was not obtained. Therefore, we divided the remaining 200 samples into others. Further, we compared the distribution of samples of different subgroups in the high-low-risk group, and no significant difference was observed (Table 2, *p* = 0.533).

**TABLE 2:** Subtypes based on etiology of the TCGA HCC cohorts.

	High_risk	Low_risk	*p*-value
HBV	54	50	
HCV	24	25	0.533
NASH	6	12	
Others	101	99	

### Calculation of mRNAsi and DNAsi

We use one Class Linear Regression (OCLR) to quantify the stemness of tumor samples. Two stemness indices were constructed from stem cell transcriptome, methylation group, and multi-platform data: mRNA expression-based stemness index (mRNAsi) represents gene expression, and epigenetically regulated-mRNAsi (DNAsi) measures epigenetics characteristics of stem cells. A stemness index (si) is a measure of stemness ranging from low (0) to high (1).

### Evaluation of drug sensitivity

By developing regression models from cell line and gene expression profiles of Genomics of Drug Sensitivity in Cancer (GDSC), the pRRophetic algorithm predicted drug maximum 50% inhibitory concentration (IC50): (www.cancerrxgene.org/).

### Statistical and bioinformatics analyses

All analyses and graphs were constructed using R v4.1.1. We calculated the gene signature’s inter-gene correlations using the Pearson correlation test. The training set (TCGA cohort) and two test sets (ICGC and GEO cohorts) were divided into high- and low-risk groups based on the median value of the E2F target-related risk score. Group differences were calculated using the *t*-test and Chi-square test. The survival distribution and gene expression patterns of HCC patients were visualized using scatter plots and heat maps. Kaplan-Meier survival analysis determined whether high- and low-risk groups had distinct prognoses. A time-dependent ROC curve was calculated with R package time ROC. To test the independent predictability of the gene signature, the R package “survival” was used to run multivariate Cox stepwise regression models.

We used GSEA v4.1.0 to examine whether the E2F target pathway was activated in different risk categories. The Gene Ontology (GO) and Kyoto Encyclopedia of Genes and Genomes (KEGG) databases were used to explore the functions of the DEGs in high- and low-risk groups (Subramanian et al., 2005).

The CIBERSORT algorithm (https://cibersortx.stanford.edu/) and the immunophenoscore (IPS) were used to evaluate immune cells infiltrated in high- and low-risk groups (Newman et al., 2015; Charoentong et al., 2017). Finally, we used CellMiner to find new medications and biological targets based on gene-drug sensitivity (https://discover.nci.nih.gov/cellminer/home.do) (Reinhold et al., 2012).

## Results

### Prognostic role of E2F target pathway in HCC

**FIGURE 1:**
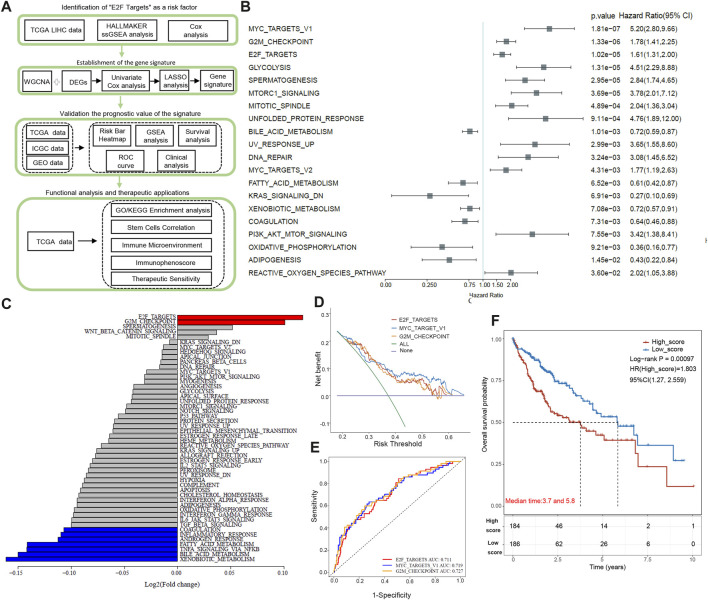
Diagram and identification of the “E2F target” pathway as a high-risk factor. **(A)** This study’s flow chart and design. **(B)** The top 20 pathways are closely associated with poor prognosis of HCC patients based on Univariate Cox regression analysis. **(C)** Comparison of the expression scores of the top 20 pathways in tumor tissue and paracancer tissue. **(D)** Decision Curve Analysis of top 3 pathways: MYC_TARGET_V1, G2M_CHECKPOINT, and E2F_TARGETs. **(E)** ROC curve analysis of top 3 pathways: MYC_TARGET_V1, G2M_CHECKPOINT, and E2F_TARGETs. **(F)** By Kaplan-Meier analysis, patients with high E2Fs scores have shorter overall survival than those with low E2Fs scores.

Figure 1A illustrates the overall workflow. Using univariate Cox regression, we calculated 50 pathways ssGSEA scores in 370 TCGA HCC samples. The top 20 pathways with statistical significance are listed in Figure 1B, among which MYC_TARGET_V1, G2M_CHECKPOINT, and E2F_TARGETs are the first three items (*p* < 0.0001). Next, we compared the expression scores of these pathways in tumor tissue and paracancer tissue. E2F target showed the most significant score difference between tumor and paracancer tissue (Figure 1C). The score of G2M_CHECKPOINT in the tumor was also higher than that in para-cancer tissue, and the difference between tumor and para-cancer was the second largest (Figure 1C). The score of MYC_TARGET_V1 in tumors was significantly lower than in adjacent tissues, as shown in Figure 1C. By applying Decision Curve Analysis (DCA), we found that MYC_TARGET_V1 performed slightly better than the other two pathways, and the evaluation effects of the E2F target and G2M_CHECKPOINT were very similar, as shown in Figure 1D. Then, we performed ROC curve analysis, and the results showed that G2M_CHECKPOINT had the highest AUC value, but the AUC values of the three channels were all above 0.71 (Figure 1E). There was no significant difference in ROC diagnostic performance among the three channels. Based on the above results, we finally choose the E2F target as the main content of this study. According to the median value (0.4072497) of the E2F target pathway, we classified HCC patients as low- and high-score groups. Kaplan–Meier survival curves showed that high-score patients have a shorter overall survival time than low-score individuals (*p* < 0.001, Figure 1F).

### Screen the candidate genes and build the E2F target signature

WGCNA co-expression algorithm identified co-expressed coding genes and modules of 370 HCC patients from the TCGA database. As a first step, we clustered the samples using hierarchical clustering and calculated the distance between each gene using the Pearson correlation coefficient. We used WGCNA to build a weight co-expression network with a soft threshold 10 to identify co-expression modules. To ensure that the network is scale-free, β= 10 is selected (Supplementary Figure S1). The expression matrix was converted into an adjacency matrix, which was then converted into a topological matrix. A hierarchical clustering method based on average linkage was employed to cluster genes, and each gene network module had a minimum of 50 genes per mixed dynamic shear tree standard. Cluster analysis was performed on the gene modules after the eigengenes of each module were calculated. Modules with relatively close distances were grouped as new modules.

**FIGURE 2:**
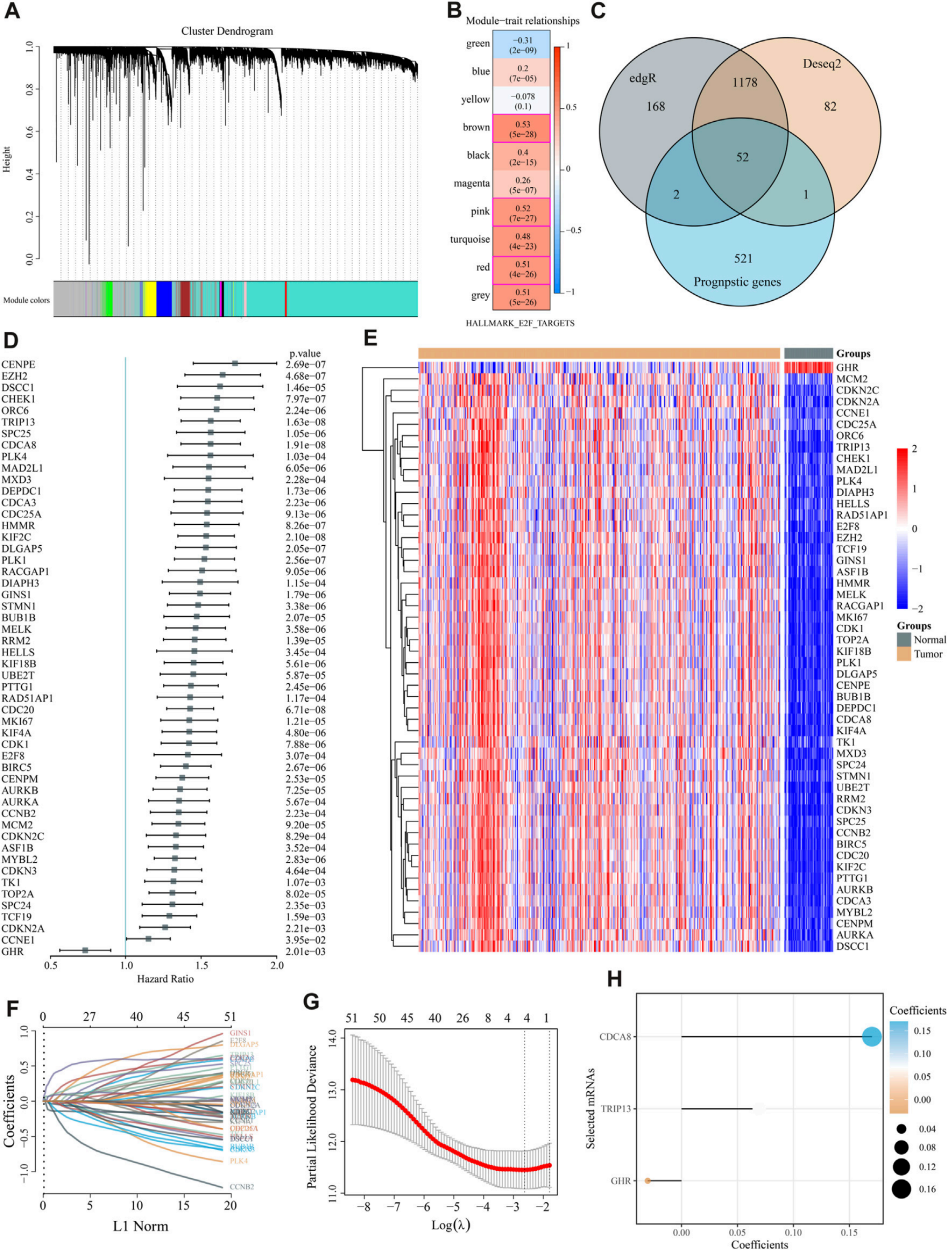
Modeling of an “E2F target” risk score. **(A)** Gene dendrograms and module colors for the WGCNA analysis. **(B)** The red, pink, and brown modules were significantly and consistently upregulated in gene ontology (GO) analysis. **(C)** Venn diagrams depicted 52 prognostic differential genes obtained by fusing DESeq, edgR, and prognostic genes. **(D)** Univariate Cox regression results for 52 genes are shown in the forest map. **(E)** The heat map reveals 52 DEGs\ between liver cancer and adjacent normal liver tissue. **(F,G)** We used LASSO Cox regression to identify the signature; the best log (λ) value was −2.63, and 3 indicators remained. **(H)** LASSO coefficients for the three genes in the signature.

When setting minModuleSize = 50, deepSplit = 3, and height = 0.25, we got a sum of 10 modules (Figure 2A). Grey modules are unaggregatable gene sets. Moreover, we analyzed the correlation between each module and each subtype and identified 609 genes with high correlation coefficients (above 0.5) with E2F, including the red (84), pink (82), and brown modules (443). These 609 genes and MSigDB E2F target pathway genes yielded 809 E2F target pathway genes. Using univariate Cox regression analysis, 576 E2F-related prognostic genes were identified (*p* < 0.05). Meanwhile, edgR and Deseq2 were used to analyze the differences between liver and para-cancer samples. A total of 1,400 DEGs (1,222 upregulated and 178 downregulated in the tumor) were identified by edgR, while 1,313 DEGs (1,100 up-expressed and 213 down-expressed) were obtained by Deseq2. These differential genes and 576 E2F-related prognostic genes revealed 52 differential prognostic genes (Figure 2C). Figure 2D exhibited the univariate Cox regression results of these 52 genes. Figure 2E shows the expression heatmap of 52 genes in HCC and adjacent liver tissue. Using the R software package glmnet, we conducted a lasso cox regression analysis. In Figures 2F,G, we analyzed the confidence interval for each lambda. Figure 2H showed that when lambda = 0.07227, there are three genes remained in the model, namely, growth hormone receptor (GHR), thyroid hormone receptor interactor 13 (TRIP13), and cell division cycle associated 8 (CDCA8). The final model formula is:

$$\text{RiskScore}=-\;0.0302*{\exp\text{⁡}}^{GHR}+0.0694*{\exp\text{⁡}}^{TRIP13}+0.1701*{\exp\text{⁡}}^{CDCA8}$$

The formula suggested that increased GHR expression is a protective factor in HCC and related to low risk, while TRIP13 and CDCA8 were associated with high RiskScore and poor outcomes. TRIP13 accelerates the mitotic process and leads to chromosome instability via playing a role in spindle assembly checkpoint and DNA repair pathways (Lu et al., 2019). High TRIP13 gene expression is found in HCC tissues, which promotes cell growth and metastasis via activating AKT/mTOR and silencing TGF-β1/smad3 pathway (Yao et al., 2018; Zhu et al., 2019). Many researchers have identified CDCA8 as a novel oncogene, which predicts a poor prognosis in HCC as well as promotes tumor proliferation via MEK/ERK, AKT/β-Catenin, and CDK1/cyclin B1 signaling (Jeon et al., 2021; Cui and Jiang, 2023). In the terms of the GHR gene, some researchers have observed its upregulation promoted HCC development (Garcia-Caballero et al., 2000; Haque et al., 2022). On the other hand, its downregulation has been noted in HCV-induced HCC and related to an unfavorable outcome (Lin et al., 2021; Abu El-Makarem et al., 2022).

We searched the clinical information of the TCGA HCC cohort (*n* = 380) from the UCSC database. As a result, 104 samples with Hepatitis B, 56 samples with Hepatitis C, and 20 samples with Non-Alcoholic Fatty Liver Disease were retrieved. Alcoholic liver disease (ALD) related content was not obtained. We divided the remaining 200 samples into others. Next, we analyzed the prognostic KM curves of the three genes in the analysis model in different subpopulations, and the results showed that GHR showed low expression and poor prognosis in HCV samples and total samples, which was consistent with the trend presented in our model (Supplementary Figures S2A–D). TRIP13 and CDCA8 only showed high expression and poor prognosis in total samples, which was also consistent with the trend shown in our model (Supplementary Figures S2A–D). Finally, we observed the expression of the three genes in different subgroups, and the results showed that the expression of GHR in different subgroups was significantly lower than that in adjacent tissues. TRIP13 and CDCA8 were significantly higher than paracancer tissues, as shown in Supplementary Figure S2E.

### A high E2F risk score predicts a poor prognosis in training group

**FIGURE 3:**
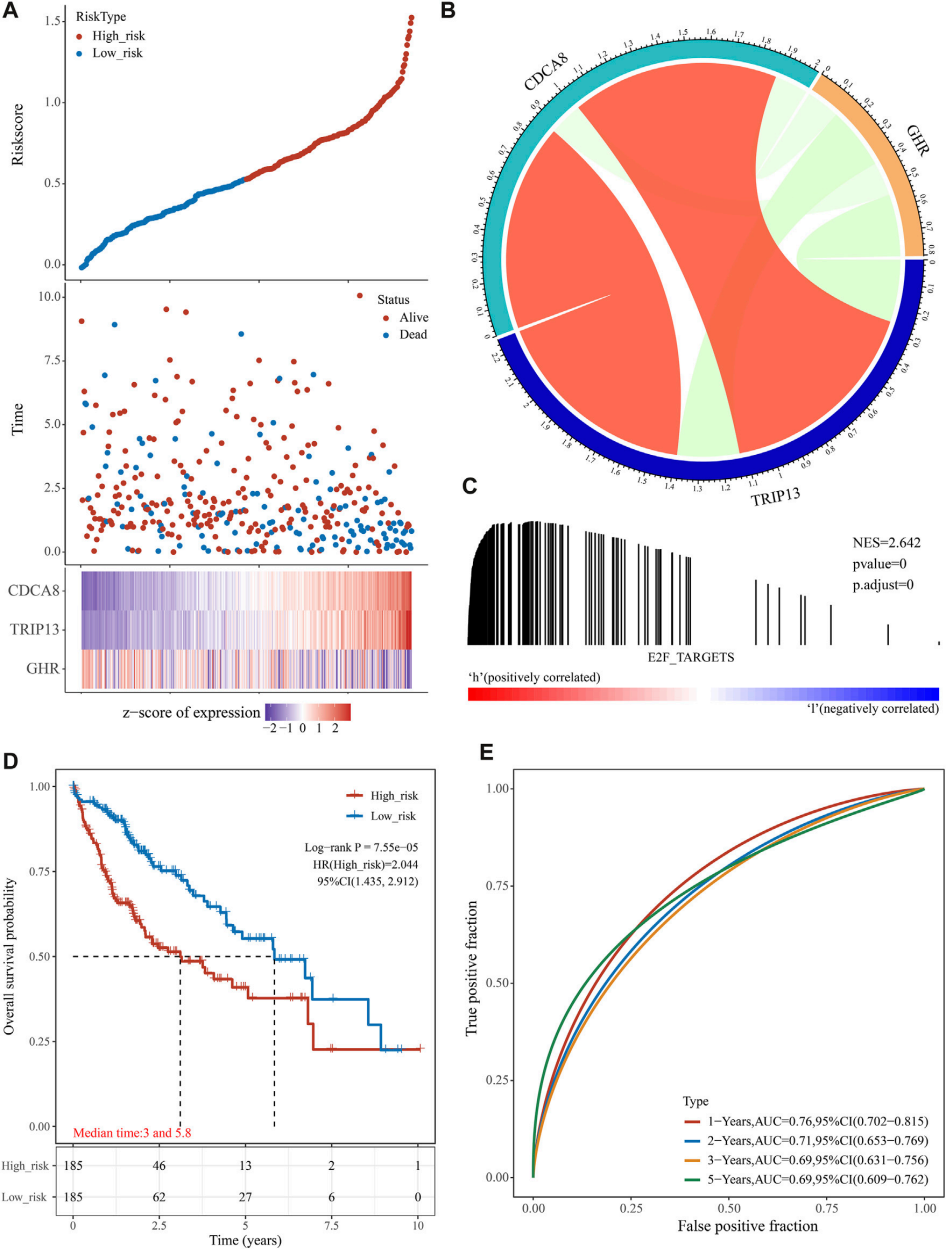
The prognostic analysis of the E2F target-related signature on TCGA cohort. **(A)** The distribution of patient risk score-survival and heat map of “E2F target”-related genes. **(B)** According to the gene correlation heat map, three genes were not highly correlated. **(C)** In the high-risk group, GSEA detected the “E2F Target” pathway activation. **(D)** A Kaplan-Meier survival analysis showed that the overall survival time was shorter in patients in high-risk groups. **(E)** ROC analysis showed that the gene signature had a high 5-year AUC, indicating a powerful predictive ability.

We generated the risk score for each training group sample and presented the RiskScore distribution (Figure 3A). Patients with a high RiskScore have a worse prognosis than those with a low RiskScore for HCC (Figure 3A). TRIP13 and CDCA8 were risk factors whose expression altered with increasing risk value, whereas high GHR expression was a protective factor linked with low risk (Figure 3B). Using GSEA, it was confirmed that genes associated with the E2F target pathway were significantly enriched in high-risk individuals (Figure 3C). We divided TCGA HCC samples into high- and low-risk groups based on the median risk score (0.528115). The KM curve displayed that the OS of the high-risk group was significantly lower than the low-risk group [Figure 3D, log-rank *p* < 0.0001, HR = 2.044 (1.435–2.912)]. Using R software package timeROC, we assessed prediction power over one, two, three, and 5 years. This risk model has a very high AUC area below the line (AUC >0.69, Figure 3E).

Two published papers have constructed E2F-related models in liver cancer, including E2F target gene characteristics of five genes constructed by Hu et al. (2022), and two gene models constructed by Wang et al. (2023), respectively. To figure out whether our new E2F target signature has an advantage, we calculated the risk score of each sample according to the formulas provided in the paper. 371 TCGA HCC samples and corresponding prognostic follow-up information provided in TCGA were used for the KM curve and AUC analysis. The results showed that the Kaplan-Meier curves of all models showed significant differences, as shown in Supplementary Figure S3A. The AUC value of the model constructed by Hu et al. (2022) was above 0.658. The AUC value of the model constructed by Wang et al. (2023) is above 0.658, as shown in Supplementary Figure S3B; Compared with the above results, we found that the AUC values of the two models are lower than ours. Also, through c-index analysis, the c-index value of our model is 0.68 (Cl 95%:0.63–0.73), and the c-index value of the model constructed by Hu et al. (2022) is 0.65 (Cl 95%: 0.63–0.71). The c-index value of the model Wang et al. (2023) constructed was 0.64 (Cl 95%:0.59–0.69). Finally, through the DCA decision curve analysis, we found that the return rate of the E2F model we built was also higher than that of the other two models, as shown in Supplementary Figure S3C.

### The prognostic value of the E2F target-related risk score was validated in the test cohort

**FIGURE 4:**
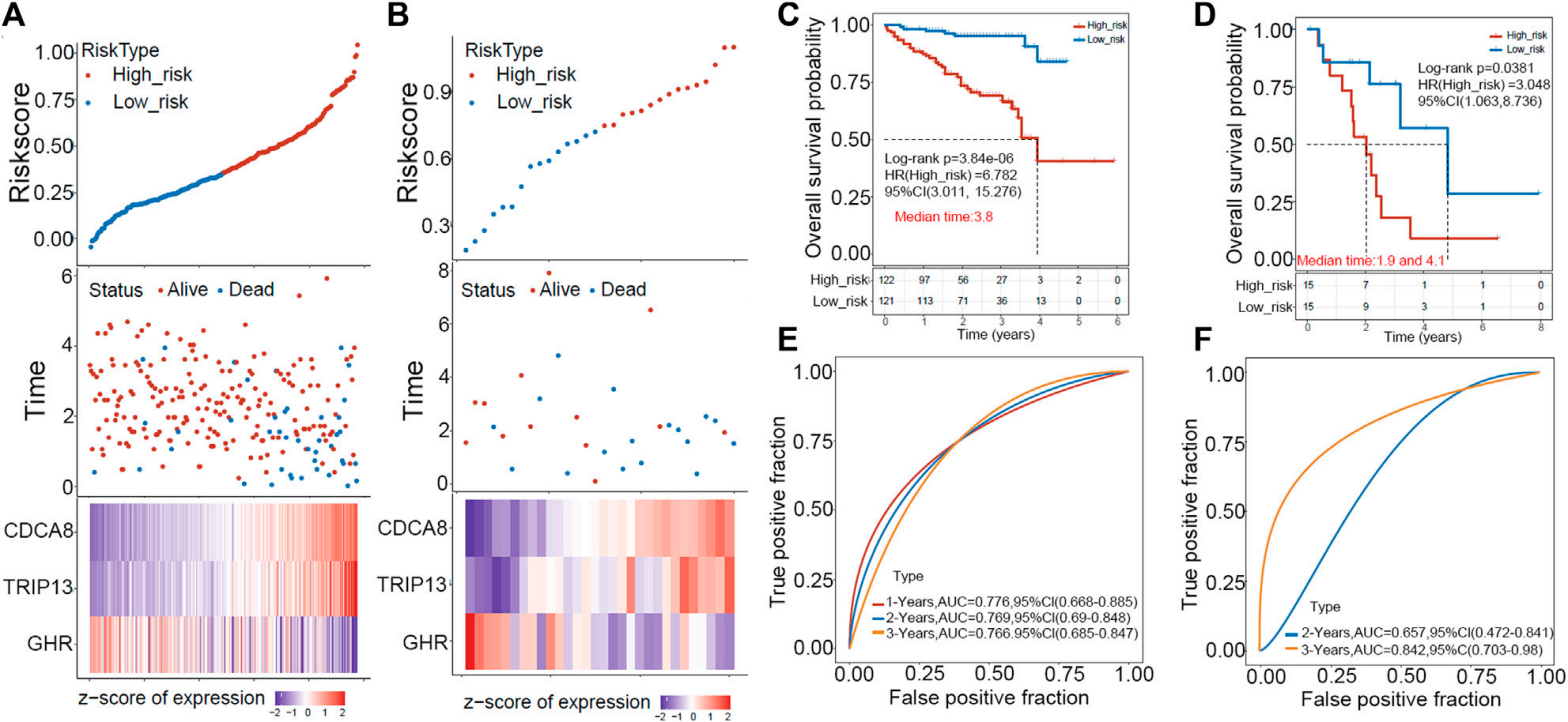
The GEO and ICGC cohorts were used to validate the prognostic signature externally. **(A,B)** A distribution map of risk score survival and a heat map of expression of “E2F target”-related genes in ICGC (left) and GEO (right) cohorts are shown. **(C,D)** In ICGC (left) and GEO (right), patients in high-risk groups displayed shorter overall survival time. **(E,F)** ROC analysis indicated a high 3-year AUC for the gene signature, suggesting its power as a predictive tool in the ICGC (left) and GEO (right) cohorts.

ICGC and GEO HCC cohorts were used as external validation sets to evaluate this risk model’s predictive value. First, the risk score of each sample was calculated in two datasets, and the RiskScore distribution of the samples was plotted as shown in Figures 4A, B. Next, we separated patients in two validation cohorts into high- and low-risk groups according to the median risk score (ICGC cohort: 0.3101617, GEO cohort: 0.7349518). Kaplan-Meier plotter revealed that patients in high-risk groups have poor prognoses within 6 years in the ICGC cohort and 8 years in the GEO cohort (Figures 4C, D). The prediction power was evaluated for one, two, three, and 5 years using the R software package timeROC. Figures 4E, F showed that the AUC area below the line is very high for the risk model, with 0.76 for the ICGC cohort and 0.657 for the GEO cohort.

### Gene signature and clinical characteristics

**FIGURE 5:**
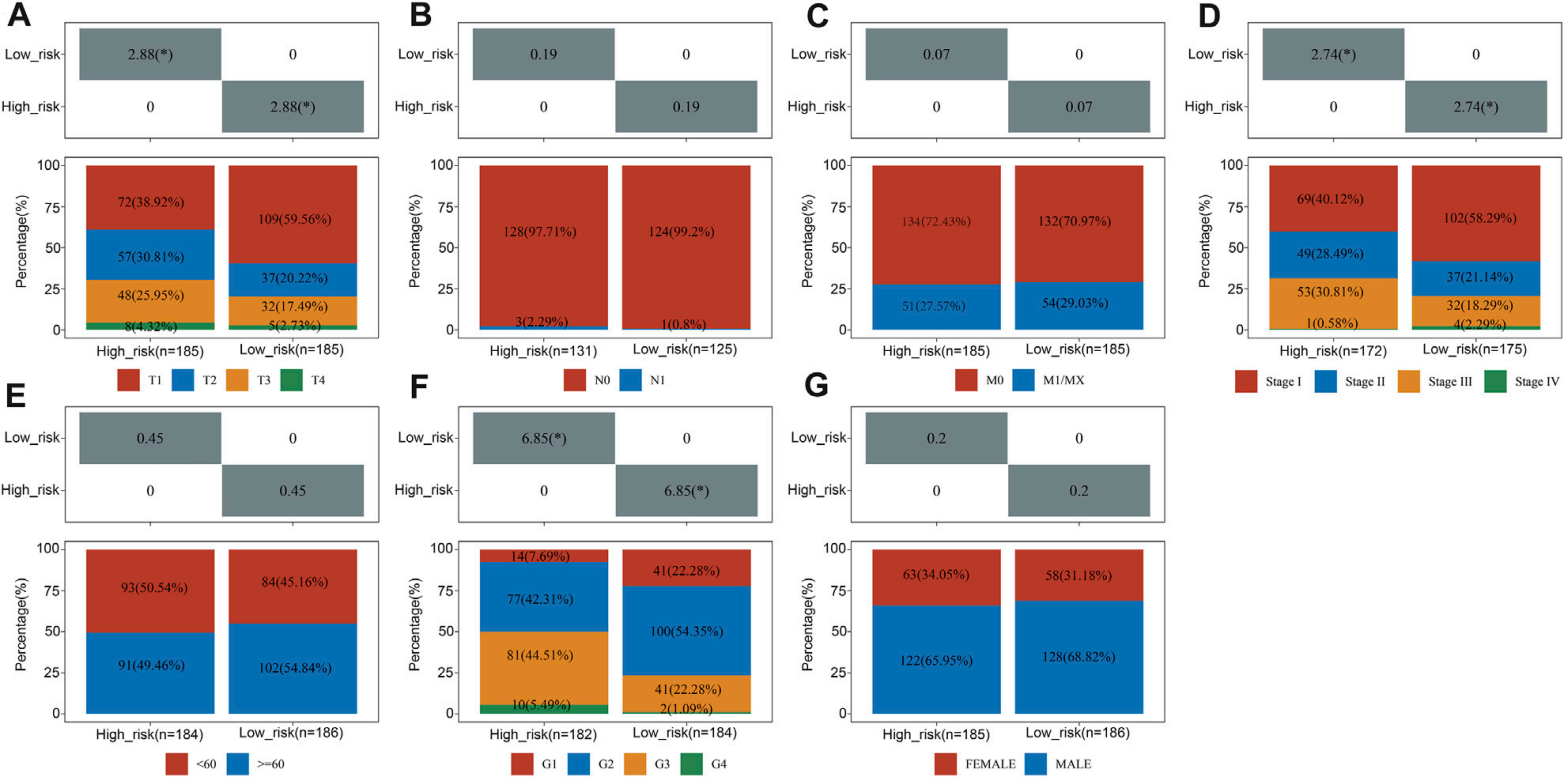
The relationship between clinical characteristics and risk groups. The correlation between the risk score and T Stage **(A)**, N stage **(B)**, M stage **(C)**, TNM stage **(D)**, sex **(E)**, differentiated degree **(F)** and sex **(G)**.

Pearson correlation analysis assessed whether risk groups and pathological characteristics were correlated. According to our findings, advanced T Stage, advanced stage, and high grade were significantly correlated with high-risk groups (*p* < 0.05, as shown in Figure 5).

### Different functions of two risk groups

**FIGURE 6:**
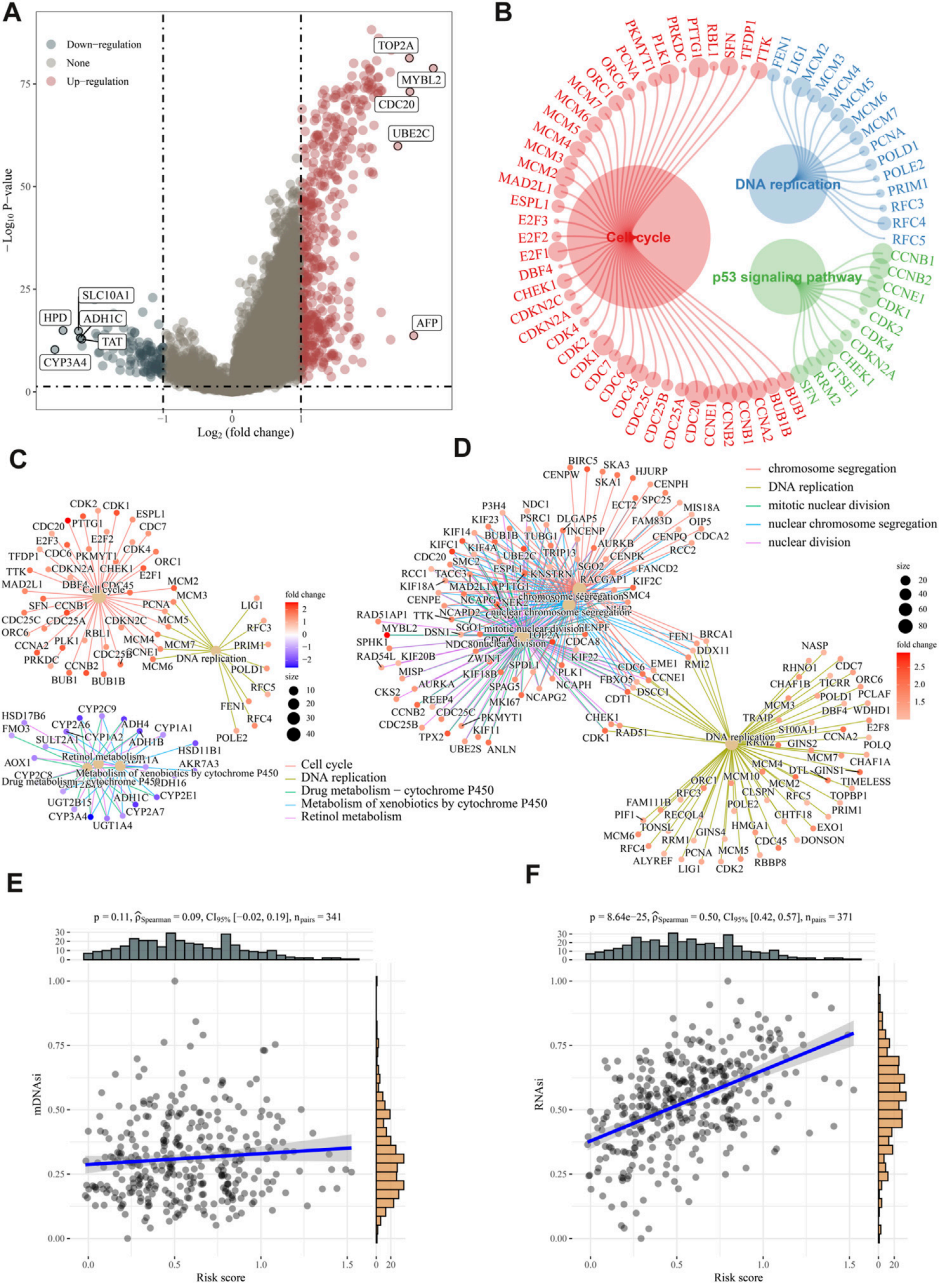
Analysis of biological function and stem cell characteristics. **(A)** DEG volcano diagrams between high- and low-risk groups. **(B–D)** According to KEGG and GO enrichment analyses, DNA replication, and cell cycle pathways were active in high-risk patients. **(E–F)** A positive correlation was found between the “E2F target” risk score and DNAsi and RNAsi.

We calculated the DEGs between high- and low-risk groups using the “limma” algorithm, and the threshold was set as *p* < 0.05. Figure 6A shows 473 upregulated and 115 downregulated genes in the high-risk group. To analyze functional enrichment in DEGs, we used R’s clusterProfiler package and parameterized the threshold to an FDR of 0.05. According to KEGG analysis, the high-risk group showed activation of the DNA replication pathway, cell cycle pathway, and p53 signaling pathway (Figures 6B, C). Based on the GO analysis, E2F targets are primarily involved in DNA replication and cell cycle checkpoints (Figure 6D). The association between risk ratings and tumour stem cell features were also evaluated. Risk score has no relationship with DNA stem index (mDNAsi) but is significantly correlated with RNA stem index (mRNAsi), as shown in Figures 6E, F.

### Immune microenvironment and therapeutic sensitivity

**FIGURE 7:**
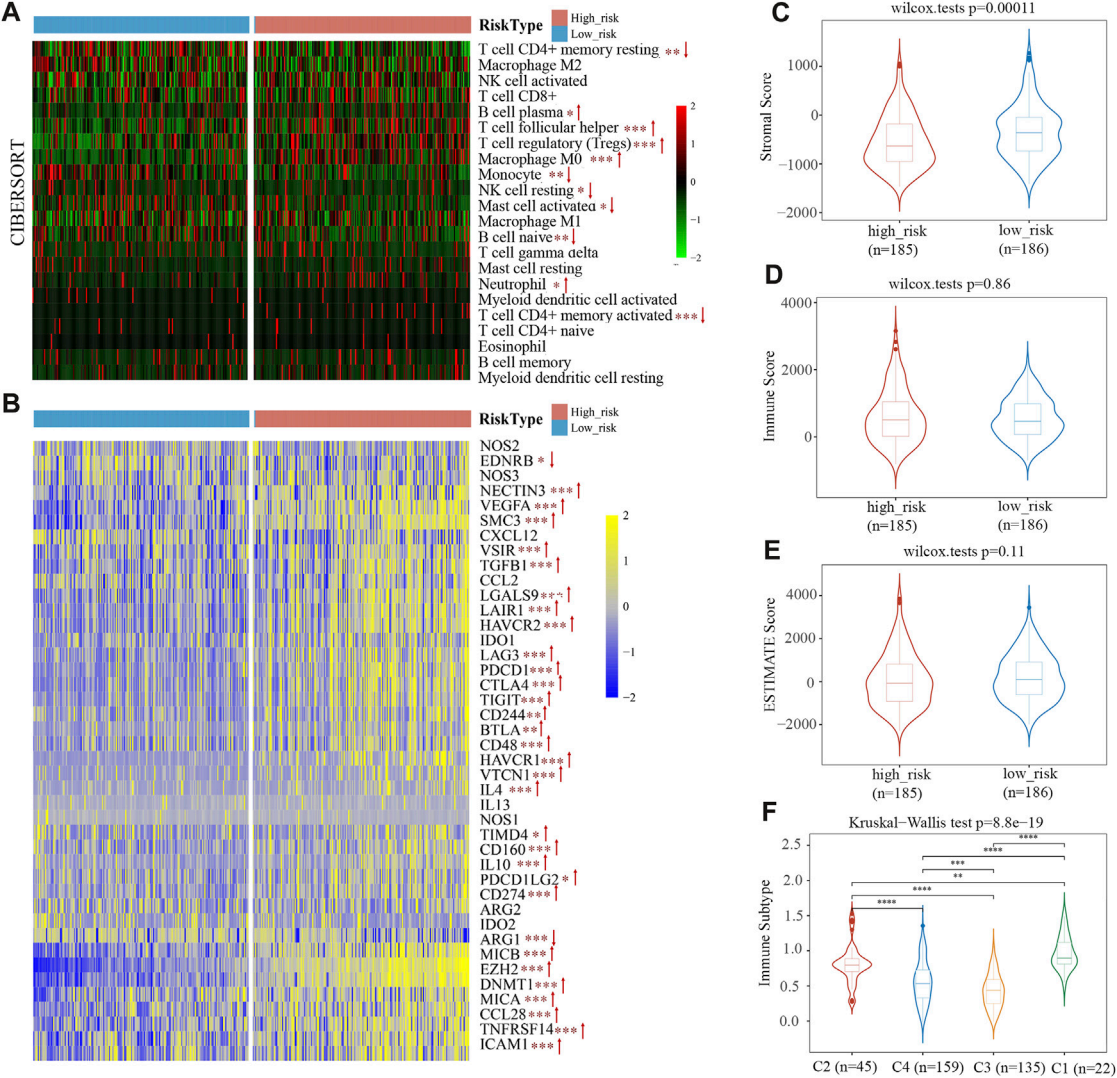
The immune cell infiltration in TCGA cohort. **(A)** Infiltration of 22 immune cells in high- and low-risk groups. **(B)** Heat map of immunosuppressor genes’ expression in two risk groups. **(C–E)** Stromal score **(C)**, immune score **(D)**, and ESTIMATE Score **(E)** between the low- and high-risk groups. **(F)** Immunotyping results indicated that the risk scores of C1, C2, and C4 types were higher than C3.

CIBERSORT was utilized as an online tool to deconvolute expression matrices of human immune cell subtypes using linear support vector regression (Newman et al., 2015). We characterized the differences of 22 immune cell infiltration between high- and low-risk groups. In the high-risk group, there were more infiltrating B cell plasma, T cell follicular helper, T cell regulatory (Tregs), macrophage M0, neutrophils, and T cell CD4^+^ memory activated within tumor tissues than in the low-risk group (Figure 7A). In contrast, the high-risk group had fewer T cell CD4^+^ memory resting, monocyte, NK cell resting, mast cell activated, B cell naïve infiltrated compared to the low-risk group (Figure 7A). According to the CIBERSORT algorithm, the high-risk group displayed immunosuppressive infiltration and a lack of immunoactive cells. Moreover, we analyzed the expression levels of 41 immune suppressor genes from the tumor immunophenotype database (TIP, http://biocc.hrbmu.edu.cn/TIP/index.jsp). Results revealed that 31 genes out of 41 (75.6%) were significantly upregulated in the high-risk group, including programmed cell death 1 (PDCD1), hepatitis A virus cellular receptor 1/2 (HAVCR1/2), T cell immunoreceptor with Ig and ITIM domains (TIGIT), indoleamine 2,3-dioxygenase 1 (IDO1), enhancer of zeste 2 polycomb repressive complex 2 subunit (EZH2), and DNA methyltransferase 1 (DNMT1) (Figure 7B).

ESTIMATE predicts tumor purity and infiltrated matrix/immune cells in tumor tissue based on ssGSEA. Estimate score (tumor purity), stromal score (matrix in tumor tissue), and immune score (infiltration of immune cells in tumor tissue) are the three main scores derived from ESTIMATE. Figure 7C shows the proportion of stromal scores is significantly lower in a high-risk group than in the low-risk group. Immune and ESTIMATE Scores had no obvious difference between the two groups (Figures 7D, E). The results suggest that the stromal component of TME is more suitable for distinguishing high-risk from low-risk patients.

The TCGA data was used by Thorsson et al. (2018) to identify six immune subtypes: C1 (Wound Healing), C2 (IFN-γ Dominant), C3 (Inflammatory), C4 Lymphocyte Depleted), C5 (Immunologically Quiet), and C6 (TGF-β Dominant). The risk score of C1, C2, and C4 types with poor prognoses was higher, and C3 types with good prognoses were primarily found among patients with low-risk scores (Figure 7F). This phenomenon indicated that the immune subtype and risk score are related.

### High-risk and low-risk groups’ drug resistance and sensitivity

**FIGURE 8:**
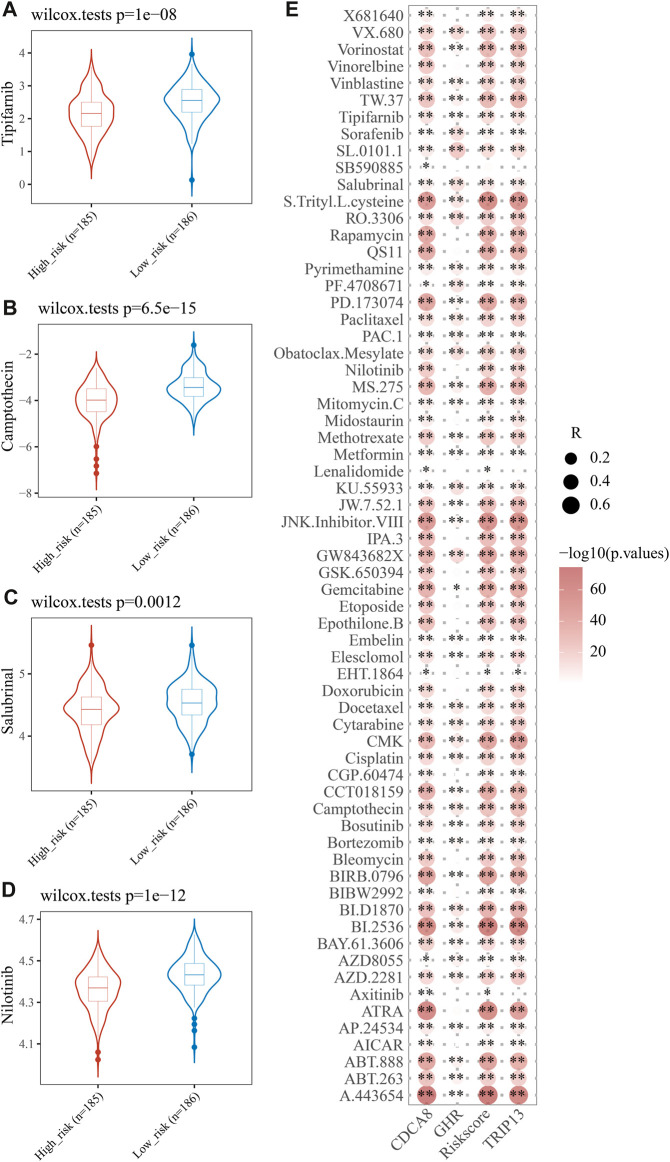
Analysis of therapeutic effect and drug sensitivity. IC50 distributions of four drugs in the high- and low-risk group, Tipifarnib **(A)**, Camptothecin **(B)**, Salubrinal **(C)**, and Nilotinib **(D)**. **(E)** Correlation analysis of three genes in the signature and common drugs used to treat HCC.

Drug resistance often emerges during cancer treatment, leading to poor efficacy and unfavorable outcome of HCC. To test E2F risk models in chemotherapy, we predicted the IC50 of 138 medicines in high- and low-risk patients using the pRRophetic algorithm. A total of 65 drugs showed significant variations between high-risk and low-risk patients, with Tipifarnib, Camptothecin, Salubrinal, and Nilotinib being more sensitive to high-risk groups. (Figure 8A, *p* < 0.05). Moreover, we found that most 65 drugs showed a significant correlation between their IC50s and risk scores (Figure 8B). The above results suggest that the E2F risk model can help HCC patients choose chemotherapy medicines based on clinical drug effects.

## Discussion

The Retinoblastoma gene (Rb) was the first tumor suppressor gene cloned and sequenced (Goodrich et al., 1991). The transcription product of the Rb gene is about 4.7 kb, and the expression product is P105-Rb, a 928 amino acid protein with a molecular weight of about 105 kDa (Classon and Harlow, 2002). P105-Rb has two states: phosphorylation and dephosphorylation. Phosphorylation is inactive, and dephosphorylation is active (Gubern et al., 2016). Dephosphorized p105 inhibits cell proliferation by binding to the transcription factor adenoviral early region 2 binding factors (E2Fs) (Dimaras et al., 2015). E2F stimulates DNA replication enzyme gene transcription. When de-phosphorized p105 binds to E2F, it inactivates E2F. Rb protein is phosphorylated by Cyclin-CDKs (CyclinD/CDK4 or CyclinE/CDK2) during the G1 phase. p-Rb releases its binding E2F to increase the transcription of Cyclin (cyclin) and CDK proteins, which causes the cell to enter the S phase from G1 phase (Zhou et al., 2022).

Researchers have characterized eight members of the mammalian E2F family, namely, E2F1-E2F8 (DeGregori and Johnson, 2006). E2F1, E2F2, E2F3, E2F4, and E2F8 are shown to be upregulated human HCC and promote cancer progression (Palaiologou et al., 2012; Liu et al., 2003a; Liu et al., 2003b; Deng et al., 2010; S et al., 2021). Cell cycle-dependent E2F transcription factors govern target gene transcription. Several E2F target genes are tightly connected, which may help predict tumor prognosis. An E2F target gene signature, formed by MDX3, PLK1, EPHA10, and KIF4A, exhibited a stronger predictive power than existing signatures in prostate cancer (Xia et al., 2022). Hu et al. (2022) established an E2F target gene signature composed of five genes (HN1, KIF4A, CDCA3, CDCA8, and SSRP1) and found it is significantly related to the prognosis of hepatocellular carcinoma. Two-E2F (E2F2 and E2F5) prognostic signature was built by Wang et al. (2023), and they estimated immune infiltration levels for patients in different risk groups. In the present study, we verified that our E2F risk model is superior to the two signatures mentioned above in predicting the overall survival of HCC patients.

Three E2F target-related genes provide a new gene signature for predicting HCC prognosis and aiding clinical decision-making. The risk score model composed of GHR, TRIP13, and CDCA8 could predict the prognosis of HCC accurately. Two external cohorts of patients from ICGC and GEO databases confirmed its prediction power. Functional enrichment analysis showed that high-risk groups were more active in DNA replication, cell cycle, p53 signaling pathway, and stem cell features.

Nowadays, anti-programmed death receptor-1 (PD-1), anti-programmed death ligand 1 (PD-L1), and anti-cytotoxic T-lymphocyte antigen-4 (CTLA-4) mAbs are among the most used immune checkpoint inhibitors in advanced HCC cases (Herbst et al., 2014). To our comfort, combining immunotherapy strategies, like anti-PD-1/PD-L1 mAbs plus anti-VEGF mAbs, TKIs, or anti-CTLA-4 mAbs, can overcome drug resistance and extend overall survival (Pinter et al., 2023). ICIs are innately resistant in 30% of HCC patients. Novel and effective biomarkers are needed to identify immunotherapy candidates. Our study used CIBERSORT to estimate immune cell infiltration. There were more immunosuppressive cells in the high-risk group than in the low-risk group, like infiltrating B cell plasma, T cell follicular helper, T cell regulatory (Tregs), macrophage M0, neutrophils, and T cell CD4^+^ memory activated. Similarly, immunosuppressive genes were upregulated. A well-designed and combined immunotherapy strategy is needed for the high-risk group due to their immunosuppressive microenvironment (Rimassa et al., 2023).

The present study found increased GHR expression to be a protective factor in HCC and related to low risk. However, according to the concrete biological context, GHR may play the dual role of inhibiting and promoting cancer. GHR downregulation was considered an independent predictor for worse outcomes in HCC (Liu et al., 2003c; Abu El-Makarem et al., 2022; He et al., 2022). In contrast, several researchers considered GHR a tumor promoter and a potential therapeutic target in HCC. Using gene knockout mice, researchers found that most Ghr+/+ and Ghr+/-mice developed HCC in response to DEN, but not the Ghr−/− mice (5.6%) (Haque et al., 2022). Scholars from Texas MD Anderson Cancer observed that tumor cells exhibited slower growth and overcame sorafenib resistance by blocking GHR with pegvisomant *in vitro* (Kaseb et al., 2022). GH inhibition downregulates ABC transporters and sensitizes HCC allografts to sorafenib (Basu et al., 2022). We noted that GHR’s downregulation is associated with HCV-induced HCC (Lin et al., 2021; Abu El-Makarem et al., 2022). We believe that rhGH and antagonists should be cautiously used in HCC patients before we fully understand the relationship between GHR and HCV virus-related HCC. High TRIP13 and CDCA8 expression predicts a favorable outcome in HCC (Zhang et al., 2009; Yao et al., 2018). Knockdown of TRIP13 and CDCA8 inhibited HCC growth and metastasis by impeding cell cycle and proliferation (Zhu et al., 2019; Chen et al., 2023).

There are several limitations to this study. Despite modern bioinformatic analysis tools, E2F target genes’ role in HCC growth and metastasis has not been explored. To answer this question, solid experiments are to unveil potential molecular pathways and their intracellular effect.

Nevertheless, new inspirations and ideas were introduced by the present study. First, we created an E2F target-related gene signature that accurately predicted HCC prognosis. Second, the E2F target pathway may have tumor-promoting effects on HCC progression based on function-enriched and immune infiltration analyses. In conclusion, we furnished HCC patients with possibly treatable target genes and responsive medicines.

## Conclusion

This study aimed to generate a signature associated with the E2F target that may be utilized to predict the prognosis of HCC. Moreover, possible pathway and mechanisms to understand how the E2F target pathway promotes tumor growth and progression of HCC have been analyzed. With the advances provided in this study, a molecular diagnosis and tiered treatment of HCC patients may be feasible. Our discovery also provides a theoretical basis for researchers to examine E2F target genes as potential HCC treatment targets.

## Data Availability

The original contributions presented in the study are included in the article/Supplementary Material, further inquiries can be directed to the corresponding author.
