# Supplementary material for: Three E2F target-related genes signature for predicting prognosis, immune features, and drug sensitivity in hepatocellular carcinoma
Source: Front Mol Biosci. 2023 Oct 3;10:1266515. doi: 10.3389/fmolb.2023.1266515 (PMC10579819; doi:10.3389/fmolb.2023.1266515)
Supplement: Supplementary file 1 [file Table1.DOCX]

Supplementary Figure 1 β=10 is selected to ensure that the network is scale-free.

Supplementary Figure 2 Prognosis of GHR, TRIP13, and CDCA8 in different subpopulations. A: Prognostic KM curve of model genes in HBV subpopulation; B: Prognostic KM curve of model genes in HCV subpopulation; C: Prognostic KM curve of model genes in NASH subpopulation; D: Prognostic KM curves of model genes in other samples; E: Expression of model genes in different subpopulations.

Supplementary Figure 3 The comparison with other E2F models. A: Prognostic KM curve of the model constructed by Hu W and Wang L et al. B: Hu W and Wang L et al. constructed ROC curves and AUC values of models 1, 2, 3, and 5 years; C: DCA decision curves of 3 models.
