# Supplementary figures and images for: Three E2F target-related genes signature for predicting prognosis, immune features, and drug sensitivity in hepatocellular carcinoma
Source: Front Mol Biosci. 2023 Oct 3;10:1266515. doi: 10.3389/fmolb.2023.1266515 (PMC10579819; doi:10.3389/fmolb.2023.1266515)

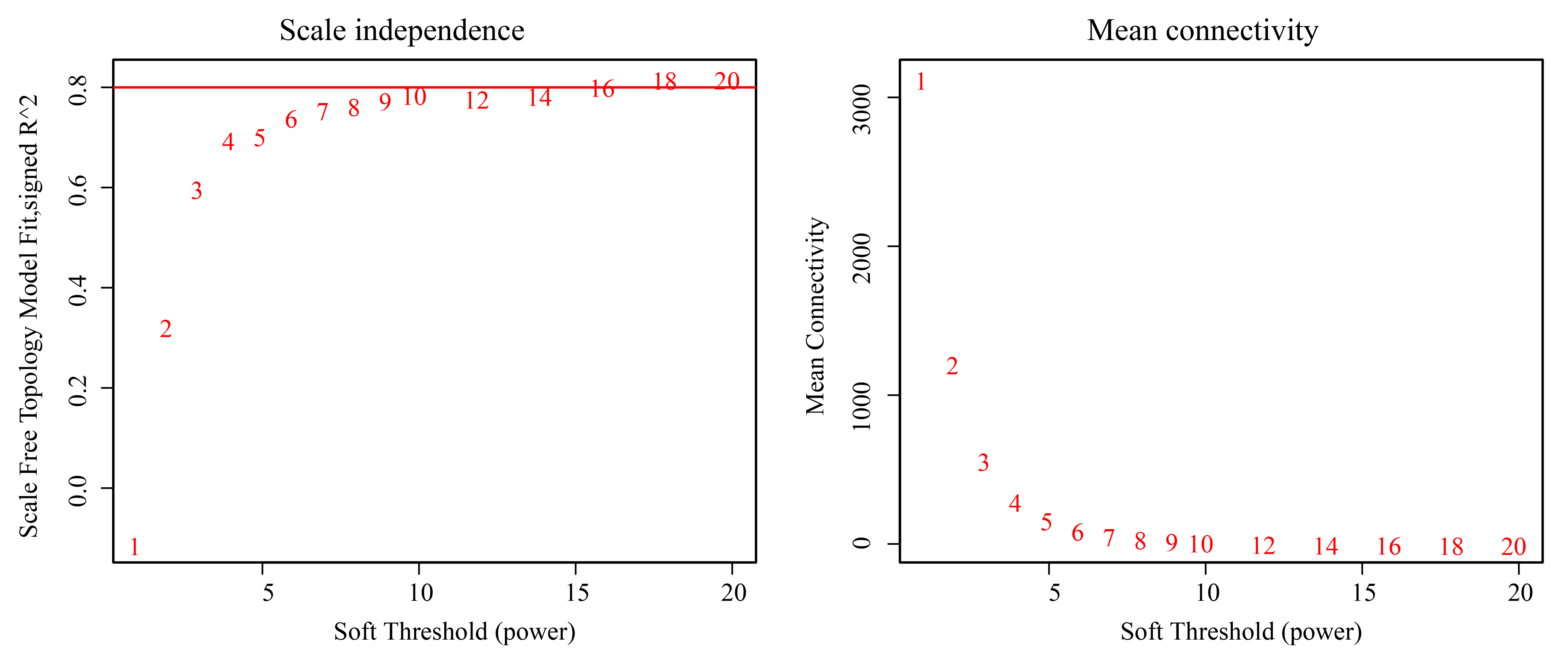

Supplement: Supplementary file 4 [file Image1.TIF]
